# Isolation and characterization of different promising fungi for biological waste management of polyurethanes

**DOI:** 10.1111/1751-7915.13346

**Published:** 2018-12-27

**Authors:** Audrey Magnin, Lucie Hoornaert, Eric Pollet, Stéphanie Laurichesse, Vincent Phalip, Luc Avérous

**Affiliations:** ^1^ BioTeam/ICPEES‐ECPM UMR CNRS 7515 Université de Strasbourg 25 rue Becquerel 67087 Strasbourg Cedex 2 France; ^2^ Soprema 14 rue de Saint‐Nazaire 67025 Strasbourg Cedex 1 France; ^3^ Univ. Lille, INRA, ISA, Univ. Artois, Univ. Littoral Côte d'Opale EA 7394 – ICV – Institut Charles Viollette 59000 Lille France

## Abstract

As a highly resistant polymer family, polyurethanes (PU) are responsible for increasing environmental issues. Then, PU biodegradation is a challenging way to develop sustainable waste management processes based on biological recycling. Since the metabolic diversity of fungi is a major asset for polymer degradation, nearly thirty strains were isolated from sampling on six different PU wastes‐containing environments. A screening of the fungi on four thermoplastic PU (TPU) with different macromolecular architectures led to the selection of three strains able to use two polyester PU as sole carbon source: *Alternaria* sp., *Penicillium* section Lanata‐Divaricata and *Aspergillus* section flavi. Weight loss, FT‐IR, Scanning Electron Microscopy and Size Exclusion Chromatography analyses revealed that these three fungi degrade slightly and similarly a fatty acid dimer‐based TPU while variability of degradation was noticed on a polycaprolactone‐based TPU. On this last TPU, robust analysis of the degraded polymers showed that the *Penicillium* strain was the best degrading microorganism. Membrane enzymes seemed to be involved in this degradation. It is the first time that a strain of *Penicillium* of the section Lanata‐Divaricata displaying PU biodegradation ability is isolated. These newly discovered fungi are promising for the development of polyester PU waste management process.

## Introduction

Material longevity associated with massive scale production makes synthetic polymers among the most abundant pollutants of our century. Studies attesting of their negative impacts on marine and terrestrial ecosystems are published at an increasing rate (Jambeck *et al*., 2015; Cao *et al*., 2017; Chae and An, 2017). Polyurethanes (PU) as the 6th polymer with 18 Mt produced in 2016 (Furtwengler *et al*., 2017), are among the most pollutant polymers (Reddy *et al*., 2006). For instance, among the 70 plastic samples collected on a beach 39 were identified as PU (Turner and Lau, 2016). PU are intended for long‐term applications: foams for construction and automotive industries, elastomers for sportswear or medical devices, coatings and sealants (Reulier and Avérous, 2015). They are thus designed to resist environmental conditions. Management of PU products end‐of‐life is currently inappropriate with most of PU wastes going to landfill and existing solutions such as incineration are not economically and environmentally satisfying. Thus, more high value added and sustainable solutions for PU waste treatment are urgently needed.

Bioremediation is a general term to qualify the action of depollution using a biological entity while biological recycling implies obtaining valuable products from waste also using biological means. Polymer biodegradation is the basis for both processes (Wierckx *et al*., 2015). Briefly, microorganisms secrete extracellular enzymes able to depolymerize the macromolecules leading to the formation of oligomers and monomers. Then, these molecules can be assimilated by microorganisms and metabolized for biomass and energy production (Lucas *et al*., 2008). Degradation can also be performed by a single enzyme from microbial, vegetal or mammalian origins (Marchant *et al*., 1987; Tokiwa *et al*., 1988; Brzeska *et al*., 2015). Bioremediation and biological recycling are attractive ways to fight against plastic pollution because of their low costs and environmental impacts (Azubuike *et al*., 2016).

The biodegradation of PU materials is increasingly studied. PU are mainly synthesized by the reaction between polyols and polyisocyanates. The polyol is generally a polyester or a polyether. The micro‐organization of PU with soft and rigid segments is one of the main key towards high‐performance materials (Carré *et al*., 2016). The building blocks involved in PU synthesis control the final macromolecular architecture, the crystallinity and the molar mass, which are parameters highly impacting the final PU biodegradability. For instance, polyester‐based PU are known to be more biodegradable than polyether ones due to the hydrolysable ester bonds (Cregut *et al*., 2013).

Degradation of PU with filamentous fungi is described as more efficient than with bacteria due to outstanding enzymatic arsenals and abiotic features linked to filaments formation (Barratt *et al*., 2003; Lucas *et al*., 2008). Several types of PU materials such as polyether urethane foam (Alvarez‐Barragan *et al*., 2016), thermoplastic polyester (Khan *et al*., 2017) and PU waterborne dispersion of polyester PU (Russell *et al*., 2011) have already been described as sensitive to fungal biodegradation.

Bottlenecks in PU biodegradation are the evaluation of the degradation with efficient techniques and the understanding of the degradation mechanisms. For instance, the majority of the microorganisms were tested on commercial model PU such as Impranil^®^, a waterborne polyester PU dispersion (Rowe and Howard, 2002; Gautam *et al*., 2007). This polymer is a milky solution presenting the particularity of becoming clearer when hydrolysed. Although Impranil^®^ is largely studied because convenient for screening approaches, its chemical architecture is very specific and far to be representative of most of the common PU. Then, Impranil^®^ is not a perfect model. When more complex PU substrates are used, a significant lack of robust methods of analysis is noticeable (Urgun‐Demirtas *et al*., 2007; Ibrahim, 2009). Degradation analysis must couple overall degradation evaluation with, for example weight loss measurement and polymer surface analysis. A sufficient number of repetitions must also be considered.

In this publication, we achieved a screening of fungal strains isolated from PU‐containing environments on several tailor‐made TPU substrates, different models with different macromolecular architectures which are much more representative of the conventional PU, compared to Impranil^®^. Degradation of these model TPU represents a step towards the degradation of real and largely produced PU such as foams. Strains of fungi presenting the best degradation capacities were then identified. Analysis of the degraded polymers and enzymatic assays on the culture supernatants were then performed to suggest degradation mechanisms.

## Results

### Screening and identification of the fungi

Strains isolated were named according to the type of agar plate used for isolation (I = Impranil + PDA, P = PDA, MMI = Impranil + MM and MMP = MM) followed by a digit indicating the origin of the sample (see materials and methods). Twenty‐seven strains were isolated. Three were yeast shaped while the others were filamentous fungi.

**Figure 1:**
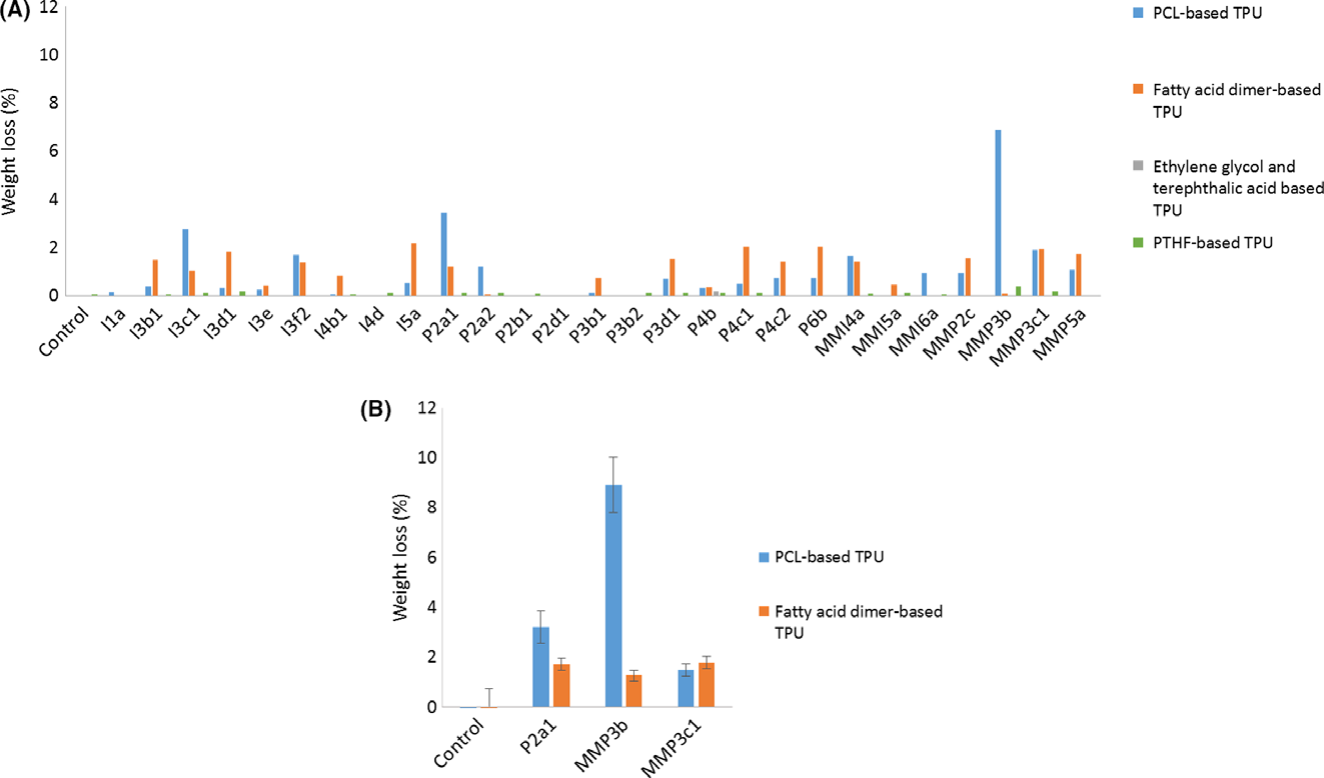
Evaluation of the weight losses on the different TPU (A) screening of the collection of fungi and (B) control of the activity of the *three* selected strains.

The collection of fungi was then screened on four TPU. No significant weight loss was detected for the ethylene glycol and phthalic anhydride‐based TPU which is a poly(ester ether)‐based TPU and for the PTHF‐based TPU (polyether‐based TPU; Fig. 1A). Evident weight losses were measured for the PCL‐based TPU and the fatty acid dimer‐based TPU incubated with 16 strains from the collection (weight loss >1% for at least one of the two polymers). The best strains were selected for further analysis. The strains P2a1 and MMP3b (Fig. 1) were selected because of weight loss higher than 3% on the PCL‐based TPU after 2 months of incubation. MMP3c1 was chosen because of the similar weight loss measured on the PCL‐based TPU and on the fatty acid dimer‐based TPU. The degradation efficiency determined with five replicates was confirmed for these three strains. They displayed weight losses coherent with those from the screening (Fig. 1B). Weight losses of 3.2%, 8.9% and 1.5% were measured for P2a1, MMP3b and MMP3c1, respectively, for the PCL‐based TPU. Weight losses of 1.7%, 1.3% and 1.8% were measured for the fatty acid dimer‐based TPU. Fungal growth was observed on the two polymers with the three strains (Fig. S1 and S2). P2a1 which was isolated from a floor mat collected in a junkyard and MMP3b and MMP3c1, isolated from a yellowish PU foam collected in a PU production plant, were thus selected as strains of interest for PU biodegradation.

**Figure 2:**
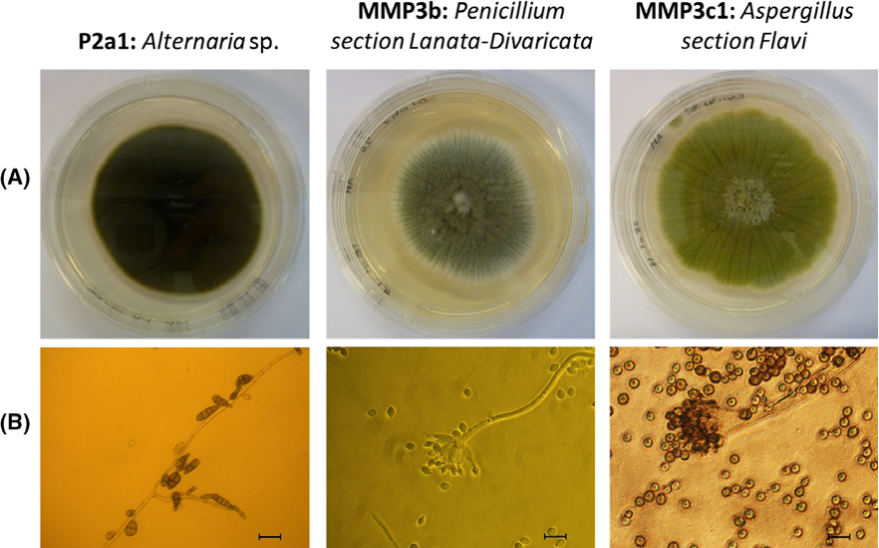
Strains selected for TPU biodegradation (A) observation on PDA after 4 days of growth at 30°C and (B) optical microscope observations of hyphae and conidiophores/conidia (Bars represent 25 μm).

The selected fungi were then identified using molecular and morphological tools. Molecular identification allows to the definition of the genus and also an orientation towards a section within the genus. The strain P2a1 was identified as member of the genus *Alternaria* (CABI deposit number: 506837). Using FASTA from EBI, the ITS sequence (NCBI accession: MH410558) matched at 98–99% identity to members of species of the genus *Alternaria* (Hou *et al*., 2016). Using the BLAST algorithm with the ‘Others’ database from NCBI, limited to sequences from type strains, this sample showed matches at 99% identity to type strains from species of *Alternaria*. There was no clear distinction between species from the sequence results; hence, identification was given at genus level. Morphological observation tends to support such identification since P2a1 conidia morphologies and growth aspects on PDA shown in Fig. 2 are similar to those of the strains of *Alternaria sp*., described in Matsumyia *et al*., (Matsumiya *et al*., 2010). The strain MMP3b ITS (NCBI accession: MH410559) sequence matches at 97.7–99.6% identity to sequences from members of *Penicillium* section Lanata‐Divaricata. This result was confirmed by partial beta‐tubulin sequencing (NCBI accession: MH594856) which also gave top matches to members of this group including 98.2% identity to sequence GU981629 from the type strain of *P. brasilianum*, which have been published in the ICPA List of *Penicillium* names and in Visagie *et al*. (2014). Morphological similarities of the conidia can be observed between Fig. 2 and the *P. brasilianum* observation from Cho *et al*. (2005) and Inokoshi *et al*. (2013). This strain was deposited in CABI collection (CABI deposit number: 506838). The ITS sequence of MMP3c1 showed 100% identity to sequence AF027863 from the type strain of *A. flavus* and to sequence EF661560 from the type strain of *A. oryzae*, both of which have been published in the ICPA list of *Aspergillus* names and in Peterson *et al*. (2017). No distinction can be made between these two species. Identification has thus been made to the section at genus level (CABI deposit number: 506839).

### Materials analysis

**Figure 3:**
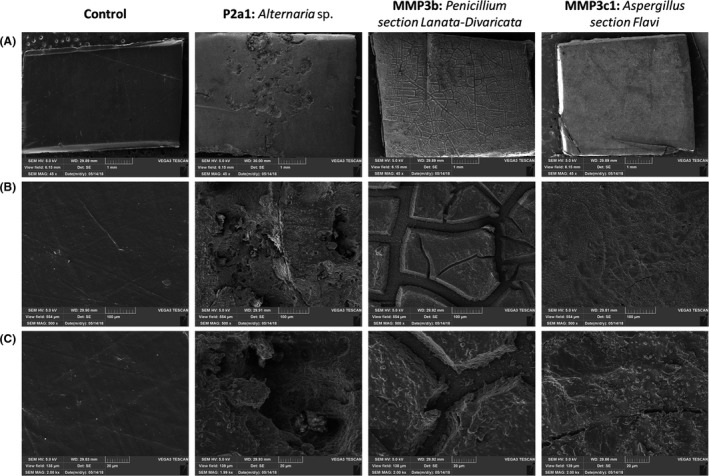
Observation of PCL‐based TPU pieces incubated 2 months at 30°C (A) magnification of 45 (bars represent 1 mm), (B) magnification of 500 (bars represent 100 μm) and (C) magnification of 2000 (bars represent 20 μm).

**Figure 4:**
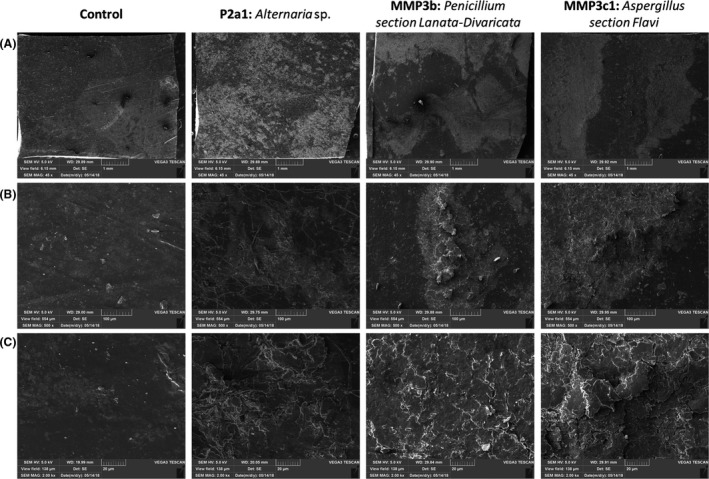
Observation of fatty acid dimer‐based TPU pieces incubated 2 months at 30°C (A) magnification of 45, (B) magnification of 500 and (C) magnification of 2000.

Several techniques were used to deepen the characterization of the degraded TPU. One replicate of each experiment was examined by SEM (Figs 3 and 4). PCL‐based TPU incubated without fungi displayed a smooth and intact surface (Fig. 3). Incubation with the strain of *Alternaria* showed deep holes disseminated in few places of the polymer surface. Incubation with the strain of *Penicillium* revealed a regular network of excavations. Finally, the strain of *Aspergillus* led to an irregular surface with shallow cracks. Fatty acid dimer‐based TPU incubated without fungi showed also an undamaged surface (Fig. 4). Whitish zones were observed when incubated with the three strains. Higher magnification on these zones revealed superimposed patches for the three degraded samples. Cracks were shallow. Only the superficial layer of the polymers was impacted.

**Figure 5:**
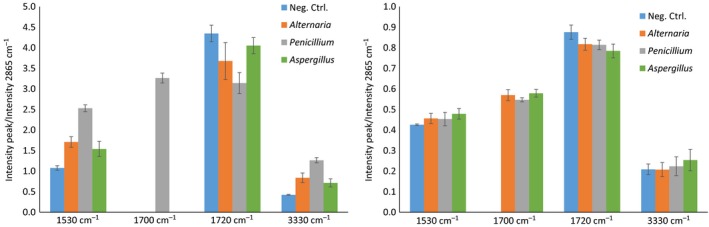
Semi‐quantitative analysis of FTIR (A) for the PCL‐based TPU and (B) for the fatty acid dimer‐based TPU.

FT‐IR was performed on all the samples to characterize the chemical modifications on TPU surfaces. Spectra, available on supplementary data (Fig. S3 and S4), allowed the identification of three zones of interest corresponding to ester or urethane bonds. A wide band between 3325 and 3340 cm^−1^ corresponds to OH and NH stretching without differentiation between these two moieties (Oprea, 2010; Spontón *et al*., 2013; Fig. S5). Peaks at 1720 cm^−1^ are attributed to C=O of an ester bond while signal at 1700 cm^−1^ can be attributed to C=O of a carboxylic acid (Elzein *et al*., 2004; Schmidt *et al*., 2017; Osman *et al*., 2018). The band at 1530 cm^−1^ is generally attributed to urethane/amide NH bending vibration in the secondary amide (Oceguera‐Cervantes *et al*., 2007; Oprea, 2010; Spontón *et al*., 2013). PCL used for the PCL‐based TPU synthesis was also separately analysed (Fig. S6). No signal appeared on this zone for this polyester. In order to perform a semi‐quantitative study, the intensity of peaks of interest was normalized by the intensity of the stable peak at 2 865 cm^−1^ corresponding to –CH_2_ stretching (Fig. 5). For the PCL‐based TPU, an increase of the signal intensities at 1530 and 3330 cm^−1^ and a decrease of the signal intensity at 1720 cm^−1^ were noticed for TPU incubated with fungi, compared to the control. A band at 1700 cm^−1^ appeared for TPU incubated with *Penicillium*. For the fatty acid dimer‐based TPU, signals intensities increase at 1530 and 3330 cm^−1^ and decrease at 1720 cm^−1^ were also observed but to a lesser extent. The peak at 1700 cm^−1^ appeared for all the TPU incubated with fungi.

All the degraded polymers were then analysed by SEC to evaluate the evolution of the molar masses distributions. Chromatograms are available on Fig. S7 and S8. Two zones can be distinguished as follows: the main polymer chain consisting in the main peak (between 4 and 7 min of retention time) and the oligomers corresponding to smaller peaks (between 7 and 8.5 min of retention). Number average molar mass (Mn), mass average molar mass (Mw), dispersity (Đ) and the percentage area of the main peak were collected in Table 1. For the PCL‐based TPU, variability between the replicates increased after fungal degradation (up to 116 000 ± 13 000 g mol^−1^) while high repeatability was observed for the negative control (125 000 ± 1000 g mol^−1^). No significant variation of Mn, Mw or Đ was noticed between PCL‐based TPU incubated with and without fungi (*P*‐value >0.05). Only a slight decrease of dispersity was observed for the strain of *Penicillium* (*P*‐value = 0.044). A decrease of the relative area of the main polymer chains was noticed for the three strains. For the fatty acid‐based TPU, a significant increase of the Mn from 35 000 for the control to 37 000, 40 000 and 38 000 was observed for the strains of *Alternaria*,* Penicillium* and *Aspergillus* respectively (*P*‐value <0.05). A significant decrease of the Mw was noticed from 120 000 g mol^−1^ for the negative control to 104 000, 108 000 and 101 000 g mol^−1^, for the strains of *Alternaria*,* Penicillium* and *Aspergillus* respectively (*P*‐value <0.05). The variation of Mn and Mw involved a high decrease of the corresponding dispersities. As for the PCL‐TPU, the relative area of the main peak also decreased significantly.

**Table 1:** SEC analysis of degraded polymers (Mn and Mw expressed as g mol^−1^)

	PCL‐based TPU				Fatty acid dimer‐based TPU			
	Neg. Ctrl.	*Alternaria*	*Penicillium*	*Aspergillus*	Neg. Ctrl.	*Alternaria*	*Penicillium*	*Aspergillus*
Mn								
Mean	58 000	55 000	55 000	57 000	35 000	37 000	40 000	38 000
SD	0	2000	6000	3000	1000	1000	1000	1000
*P*‐value		0.116	0.419	0.366		0.003	0.000	0.010
Mw								
Mean	125 000	122 000	116 000	121 000	120 000	104 000	108 000	101 000
SD	1000	8000	13 000	11 000	4000	3000	6000	4000
*P*‐value		0.439	0.148	0.267		0.001	0.017	0.001
Disp								
Mean	2.17	2.21	2.10	2.13	3.43	2.79	2.70	2.69
SD	0.01	0.05	0.04	0.08	0.06	0.05	0.10	0.14
*P*‐value		0.258	0.044	0.193		0.000	0.000	0.000
Area								
Mean	99.5%	99.0%	98.5%	99.2%	99.0%	96.3%	94.9%	94.8%
SD	0.0%	0.1%	0.5%	0.1%	0.1%	0.3%	0.3%	1.0%
*P*‐value		0.000	0.002	0.002		0.000	0.000	0.000

### Enzymatic assays

**Figure 6:**
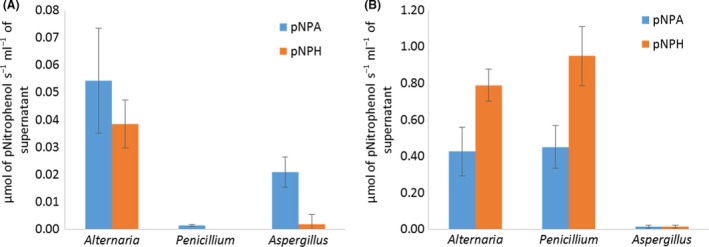
Evaluation of esterase activity with pNPA and pNPH on the supernatant of fungal cultures of (A) PCL‐based TPU and (B) fatty acid dimer‐based TPU.

Esterase activity was determined on two substrates, pNitrophenyl acetate (pNPA) and pNitrophenol hexanoate (pNPH), on the supernatant media of fungal cultures on PCL‐based TPU and fatty acid dimer‐based TPU (Fig. 6). Both chemicals release pNitrophenol after hydrolysis of the ester bonds. Measured activities were significantly higher for the fatty acid dimer‐based TPU than for the PCL‐based TPU. Enzymes of *Alternaria* and *Penicillium* produced during the degradation of the fatty acid dimer‐based TPU showed a better affinity for the substrate with six aliphatic carbons. Esterase activity of the supernatants of culture on PCL‐based TPU was the highest for *Alternaria* and null for *Penicillium*.

## Discussion

To be as close as possible to conventional PU wastes, the synthesized polymers were based on 4,4′MDI, thus providing highly resistant urethanes groups (Sabbioni *et al*., 2012). Screening on TPU only showed degradation of the two polyester PU after 2 months of incubation at 30°C with the polymers as the sole carbon source (Fig. 1). The poly(ester ether) PU and the polyether PU were already described as not degradable using efficient PU‐degrading enzymes (A. Magnin., E. Pollet., R. Perrin., C. Ullmann., C. Persillon .,V. Phalip and Avérous L. unpublished results). For the poly(ester ether) PU, it seems that the macromolecular structure makes the ester bonds not easily accessible for enzymes. The degradation of the polyester PU was also confirmed by the evaluation of the fungal growth (Fig. S1 and S2). Degradation ability of the fungi thus depends on the bonds of the soft segment of the TPU but also on the macromolecular structure. Usually, additional carbon sources such as glucose (Matsumiya *et al*., 2010; Khan *et al*., 2017) or PDB (Alvarez‐Barragan *et al*., 2016) are added to promote the microbial growth and thus, the biodegradation. Regarding the development of an efficient remediation or bio‐recycling process, working without additional carbon source such as glucose, as in our case, is a major financial gain.

Identifications of the strains were done at the genus level for the strain P2a1 which is *Alternaria sp*. and to the section for MMP3b and MMP3c1 which are respectively *Penicillium* section Lanata‐Divaricata and *Aspergillus* section flavi. *Alternaria* species have already been described for PU degradation. For example, a strain of *A. tenuissima* was reported as active on pyridine‐based polyether PU (Oprea *et al*., 2018). *Alternaria solani,* isolated from soil, showed degradation activity towards a polyester PU (Ibrahim, 2009). *Alternaria sp*. was described for the degradation of a polyether PU foam and on model molecules with a urethane bond as a sole cleavable bond (Matsumiya *et al*., 2010). *Aspergillus flavus* has been described as capable of degrading a polyester PU as sole carbon source (Mathur and Prasad, 2012) while *A. oryzae* has never been described on PU.

The strain involving the most significant weight loss on TPU is the strain of *Penicillium* section Lanata‐Divaricata. Isolated species of *Penicillium* have been described on PU degradation studies such as *Penicillium chrysogenum* (Alvarez‐Barragan *et al*., 2016), but to the best of our knowledge, no isolated strains of *Penicillium* belonging to the section Lanata‐Divaricata has been reported to degrade PU. It is thus the first time for such a strain that a PU‐degrading activity is clearly demonstrated. The best match for our strain appeared to be with *P. brasilianum. *This species is widely known as an enzyme factory, mainly for production of cellulases (Jorgensen *et al*., 2004; Jørgensen and Olsson, 2006) and feruloyl esterases (Panagiotou *et al*., 2007).

Complete analysis of the degradation systems was provided for the two polyester PU incubated with the three strains of interest. Different degradation profiles were observed and followed by weight loss, SEM, FTIR analysis and enzymatic assays for the PCL‐based TPU incubated with the three strains (Figs 1B, 3 and 4). SEC data did not provide differential results (Table 1).

Surface analysis of the PCL‐based TPU incubated with *Penicillium* by SEM revealed a specific network like a fungal mycelium's one. Moreover, Fig. 2B shows the filaments and spores of the strain of *Penicillium*, with filaments diameters from 5 to 10 μm. The network displayed rift widths slightly higher than 10 μm, strengthening the hypothesis of material excavation corresponding to filament growth on the polymer surface. The absence of extracellular esterase activity indicates that enzymes mainly responsible of the TPU degradation seem to be cell‐bound proteins. These types of enzymes have already been described on the bacterial degradation of PU (Akutsu *et al*., 1998).

Better affinity for the pNPH, ester substrate with six aliphatic carbons, was expected for the enzymes produced within the culture medium with the PCL‐based TPU as it consists in the repetition of an ester bound to a chain of six carbons. Surprisingly, none of the three strains produced enzymes with better affinity for this substrate. However, for the fatty acid dimer‐based TPU, the activity on pNPH was higher than on pNPA. This could be linked to the hydrophobic chain length of the pNPH ester group matching the one of the pendant alkyl chains of the polyester segment of this TPU.

The weight loss for the fatty acid dimer‐based TPU was low (about 1.5%) and almost similar for all three strains (Fig. 1B). Surface analysis performed by SEM and FTIR displayed similar polymer surface morphologies and similar changes in surface moieties (Figs 4 and 5). Enzymatic assays showed close esterase activities between the strain of *Alternaria* and *Penicillium* while a significantly lower esterase activity for the strain of *Aspergillus* (Fig. 6B). The mechanisms implemented by the strain of *Aspergillus* seemed to be different than the ones of *Alternaria* and *Penicillium*. Despite these differences, the three strains presented analogous impact on the fatty acid dimer‐based TPU.

In addition to strain comparison, this study allows also discussing the techniques used to evaluate the polymer degradation. For the fatty acid dimer‐based TPU, SEC analysis of the remaining solid polymer showed decreasing Mw and increasing Mn highlighting some polymers alterations. This increase in Mn values could be explained by the limited cleavage of the polymer chains along with the release of very short oligomers. These small molecules could be lost by solubilization in the surrounding medium or, in the case of fungal degradation, by their metabolization by the microorganisms. It has been reported that even a very limited loss in the lower molar mass fractions may result in a significant apparent increase in Mn values (Mohd‐Adnan *et al*., 2008). The loss of material is limited. This is highlighted by the decrease of main polymers and the evolution of the oligomers concentration (Table 1). These oligomers could correspond to the observed superimposed patches observed by SEM at the polymer surface (Fig. 4). As oligomers remained mainly associated with the polymer pieces, the weight loss was limited even when the observed degradation was significant. The same behaviour was observed on fatty acid dimer‐based TPU after 50 days of degradation at 37°c with an esterase (Magnin *et al*., unpublished). For the PCL‐based TPU, no significant change in molar mass distributions was observed on the remaining solid polymer and only low amount of oligomers were found, most probably due to their consumption by fungi. SEC appears thus to be a good technique for the analysis of polymers showing superficial ‘damages’, as a complement of the weight loss measurement, but seems less relevant for highly degraded polymers.

This work offers a clear proof of the interest of FTIR for the semi‐quantitative evaluation of TPU biodegradation. For the PCL‐based TPU, *Penicillium* showed the highest peak intensity increase at 3300 and 1700 cm^−1^ and the highest decrease at 1720 cm^−1^ while displaying the highest weight loss whereas *Aspergillus* showed the lowest intensity variations at these three wavelengths. For the fatty acid dimer‐based TPU, comparable but lower weight losses were observed with all three strains. However, FT‐IR spectra showed a slight increase in peak intensity at 3300 cm^−1^ and a decrease at 1720 cm^−1^. The intensity variation for these two peaks is thus consistent with the weight loss and thus with overall polymer degradation. However, the interpretation of the increase of the peak intensity at 1530 cm^−1^ appears trickier. Oprea (2010) suggested that an increase of this signal is related to urethane bond hydrolysis while others suggested that a decrease of this signal attests for urethane bond degradation (Oceguera‐Cervantes *et al*., 2007; Sarkar and Lopina, 2007). The mechanism and location of the hydrolysis is most likely the reason for the contradictory statements found in the literature. An esterase activity would lead to the hydrolysis of the ester bond part of the urethane group, resulting in more carbamate groups exposed and thus increased signal of secondary amide band. In the case of protease activity, the amide bond part of the urethane group would be cleaved resulting in the decrease of the corresponding band signal. The observed increase of the secondary amide signal tends to evidence that the esterase activity is predominant. But, either an increase or a decrease of this signal would attest for changes in the polymer structure. Consequently, fungal degradations have a clear impact on the urethane bond but no definitive conclusion can be made on the exact nature of this impact. This point needs additional investigations.

Identification of these three strains, and particularly the strain of *Penicillium*, is the premise for the development of bioremediation and biological recycling processes. Understanding the full metabolisms involved in the biodegradation should then be fully elucidated with, for example proteomic tools. Optimization of the conditions must be carried out to improve TPU degradation. For instance, pre‐incubation in rich media could be tested to further improve the biodegradation. More generally, systems optimization might allow to degrade other polyurethanes, such as cross‐linked ones, for example PU foam, which represent the large majority of the commercial PU. This paper lays the foundation for an effective and green solution towards relieving the burden of polyurethane waste pollution, which is in the core of one of the major and very actual concern linked to the worldwide plastic pollution.

## Experimental procedures

### Materials

#### Minimal medium preparation

Minimal medium (MM) was prepared according to Alvarez‐Barragan *et al*., with some minor modifications (Alvarez‐Barragan *et al*., 2016). Buffer with 19 mM of NaH_2_PO_4_ and 33.5 mM of K_2_HPO_4_ was supplemented by agar (15 g l^−1^ final) and HCl (1.8 mM final) and autoclaved at 121°C for 18 min. To 1 l of this buffer was added 10 ml of each of the following solutions, filtered at 0.22 μm with polystyrene filters (in this order): (i) a solution of MgSO_4_ x 7H_2_O (250 μM), (ii) a solution containing FeCl_3_x6H_2_O (147 μM), ZnCl_2_ (9 μM), CoCl_2_ (6.5 μM), Na_2_MoO_4_ x 2H_2_O (12 μM), CaCl_2_x 2H_2_O (10 μM), CuCl_2_ x 2H_2_O (13 μM), MnCl_2_ x 2H_2_O (14.6 μM), H_3_BO_3_ (12 μm) and finally, (ii) a solution of NH_4_SO_4_ (7.6 mM). Streptomycin at 60 μg ml^−1^ final was added in the MM to prevent bacterial growth.

#### Polyurethane substrate

The waterborne polyester PU dispersion used for this experiment is Impranil‐DLN^®^, kindly supplied by Covestro (Leverkusen, Germany). Four representative TPU substrates with different macromolecular architectures (i.e. PCL‐based TPU, fatty acid dimer‐based TPU, polyether‐based TPU, and poly(ester ether)‐based TPU) were synthesized. The list of polyols used for TPU syntheses is presented in Table 2. The 4,4′‐methylene diphenyl diisocyanate (4,4′MDI, Suprasec 1306 – Mn = 250.25 g mol^−1^) was purchased from Huntsman (USA). The 1,4‐butanediol (1,4‐BDO, Mn = 90.12 g mol^−1^) was purchased from Invista (USA).

**Table 2:** Polyols used for TPU synthesis

Commercial name	Supplier of the polyols	Composition	Chemical structure of the main components of the polyols
Capa 2302	Perstorp	Polyester: polycaprolactone (PCL)	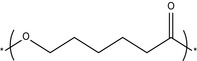
Radia 7285	Oleon	Polyester based on fatty acid dimer from rapeseed oil and short diol	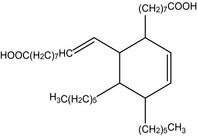
Terathane 2000	Invista	Polyether: Polytetrahydrofuran (PTHF)	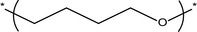
Stepanpol PD56	Stepan	Polyester based on diethylene glycol and phthalic anhydride	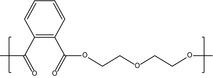

The TPU syntheses were performed into a jacketed reactor at 80°C under nitrogen gas flow. Stoichiometric conditions were used with a NCO/OH ratio of 1, using an equimolar quantity of the polyol and 1,4‐BDO. A ‘one‐step’ method was performed. The 4,4′‐MDI was warmed at 60°C and then introduced into the reactor followed by the mix of polyol and 1,4‐BDO, previously heated at 80°C. Resulting TPU material was then compressed at 145°C with a plate press (Labtech Hot Press, Samutprakarn, Thailand) to obtain thin films. They were then cut into pieces of 2 × 2 cm.

### Methods

#### Fungal isolation

Six samples likely to contain PU‐degrading fungi were collected: (i) a landfill stagnant water from a PU production plant collected in summer (Saint Julien du Sault, France), (ii) a floor mat collected in a junkyard (Oberhausbergen, France), (iii) a yellowish, old, PU foam collected in a PU production plant (Saint Julien du Sault, France), (iv) a 2‐year‐old standing water in contact with concrete slab covered by PU varnish (Strasbourg, France), (v) a PU foam collected in a discharge (Strasbourg, France) and (vi) a greenish PU foam from an old office chair (Strasbourg, France).

Solid samples were ground and sieved to obtain homogeneous fragments. Liquid samples were centrifuged, and the pellets were recovered. Each sample was dispersed until adhesion on the four media used for fungal isolation: MM and Potatoes Dextrose Agar (PDA) with and without 2 g l^−1^ of Impranil‐DLN^®^, kindly supplied by Covestro. Potatoes Dextrose Agar is a rich medium that allowed the recovery of a large number of fungi. On the contrary, MM provides extra sources of nitrogen and minerals to promote fungal growth while using the environmental sample or Impranil as carbon source. Growing fungi were successively plated until isolation. Strains isolated were finally plated on PDA for storage at 4°C.

#### Degradation assays

Degradation assays on the four synthesized TPU were performed in 40 ml sterile polystyrene flasks. Each flask was weighted beforehand. TPU pieces of 2 × 2 cm were washed twice with distilled water, once with ethanol 70% and dried overnight at room temperature under a laminar flow hood. One piece of TPU was then added into each flask and weighted. 2 ml of MM without agar were added in each flask. Medium was inoculated using fungal agar plate culture and an inoculation loop of 1 μl.

After 2 months of incubation at 30°C, cultures were stopped. Supernatants were recovered for biochemical assays, if necessary. TPU pieces were washed twice with distilled water, once with ethanol 70%, dried overnight at room temperature and finally, weighted. Negative control consisted in TPU pieces incubated in liquid MM without fungal inoculation. For the initial screening, cultures were performed with only one repetition while the confirmation step was performed with at least four repetitions for each pair of fungus/TPU.

#### Fungal identification

Selected strains were grown on PDA for 3 days at 30°C and sent to the accredited service for fungi identification of CABI (Wallingford, UK). All procedures were validated and the processing was undertaken in accordance with CABI's in‐house methods for filamentous fungi and yeasts. Strains were identified by internal transcribed spacer (ITS) rDNA sequence analysis and in one case, partial beta‐tubulin gene sequence analysis. Several algorithms were used for identification: the BLAST algorithm with the CABI Validated Sequence Database for Fungi, the FASTA algorithm with the Fungus database from EBI and the BLAST algorithm with the ‘Others’ database from NCBI, limited to type strains. Strains were deposited in CABI collection.

Nikon A1R laser confocal microscope (Nikon, Tokyo, Japan) equipped with a 100× Plan Fluor water immersion objective was used for observation of filaments and spores of fungi.

#### Characterization techniques

Scanning electronic microscopy (SEM) was used to study the evolution of the surface morphology of the degraded films. Vega‐3 (Tescan, Brno, Czech Republic) apparatus in high vacuum mode with working distances in the range of 6–8 mm and an acceleration voltage of 5 kV was used. Before examination, samples were coated with a thin layer of gold with a gold coater (Quorum Q 150 RS; Quorum Technologies, Lewes, UK).

Infrared spectroscopy was performed with a Fourier transformed infrared spectrometer Nicolet 380 from Thermo Scientific (US) used in reflection mode equipped with an ATR diamond module (FTIR‐ATR). An atmospheric background was collected before each sample analysis (32 scans, resolution 4 cm^−1^). FTIR analyses were performed on the washed samples after the 2 months of incubation.

Size exclusion chromatography (SEC) was used to determine the evolution of the polymers molar masses. TPU was dissolved in tetrahydrofuran (THF) at room temperature and filtrated at 0.2 μm with polypropylene membranes directly in vials. An Acquity APC (Waters, Milford, Massachusetts, USA) in THF at 40 °C was used for SEC analysis. Three columns (Acquity APC XT 450 Å 2.5 μm 4.6 × 150 mm, 200 and 45) were connected. 10 μl of dissolved polymer solution were injected. A run of 11 min with a flow of 0.6 ml min^−1^ was applied. A calibration curve using polystyrene (PS) standards was built for molar mass determination.

#### Enzymatic assays

The supernatants of the degradation assays were recovered and centrifuged 10 min at 10 000 g in order to remove residues of fungi. pNitrophenyl acetate (pNPA; Sigma‐Aldrich, Saint‐Louis, Missouri, USA) and pNitrophenyl hexanoate (pNPH; TCI Europe, Zwijndrecht, Belgium) were prepared at 55 mM in dimethylsulfoxyde (DMSO). In a microcuvette, 10 μl of substrate in DMSO were added to 690 μl of MM and 300 μl of centrifuged supernatant. Absorbance was measured at 415 nm at room temperature. The molar absorptivity of pNitrophenol under these assay conditions was 57.4 mM^−1^ cm^−1^. Activities were expressed as μmol of pNitrophenol s^−1^ ml^−1^ of supernatant.

## Conflict of interest

None declared.

## Supporting information


**Fig. S1**. Evaluation of the fungal growth on the PCL‐based TPU after 2 months of incubation at 30°C.

**Fig. S2**. Evaluation of the fungal growth on the fatty acid dimer‐based TPU after 2 months of incubation at 30°C.

**Fig. S3**. Absorbance spectra of FTIR analysis for PCL‐based TPU incubated 2 months at 30°C.

**Fig. S4**. Absorbance spectra of FTIR analysis for fatty acid dimer‐based TPU incubated 2 months at 30°C.

**Fig. S5**. FTIR signals attribution.

**Fig. S6**. Comparison of absorbance FTIR spectra of the PCL‐based TPU and the PCL polyester.

**Fig. S7**. SEC analysis of the PCL‐based TPU incubated 2 months at 30°C with fungi.

**Fig. S8**. SEC analysis of the fatty acid dimer‐based TPU incubated 2 months at 30°C with fungi.
