# Supplementary figures and images for: Isolation and characterization of different promising fungi for biological waste management of polyurethanes
Source: Microb Biotechnol. 2018 Dec 27;12(3):544–55. doi: 10.1111/1751-7915.13346 (PMC6465239; doi:10.1111/1751-7915.13346)

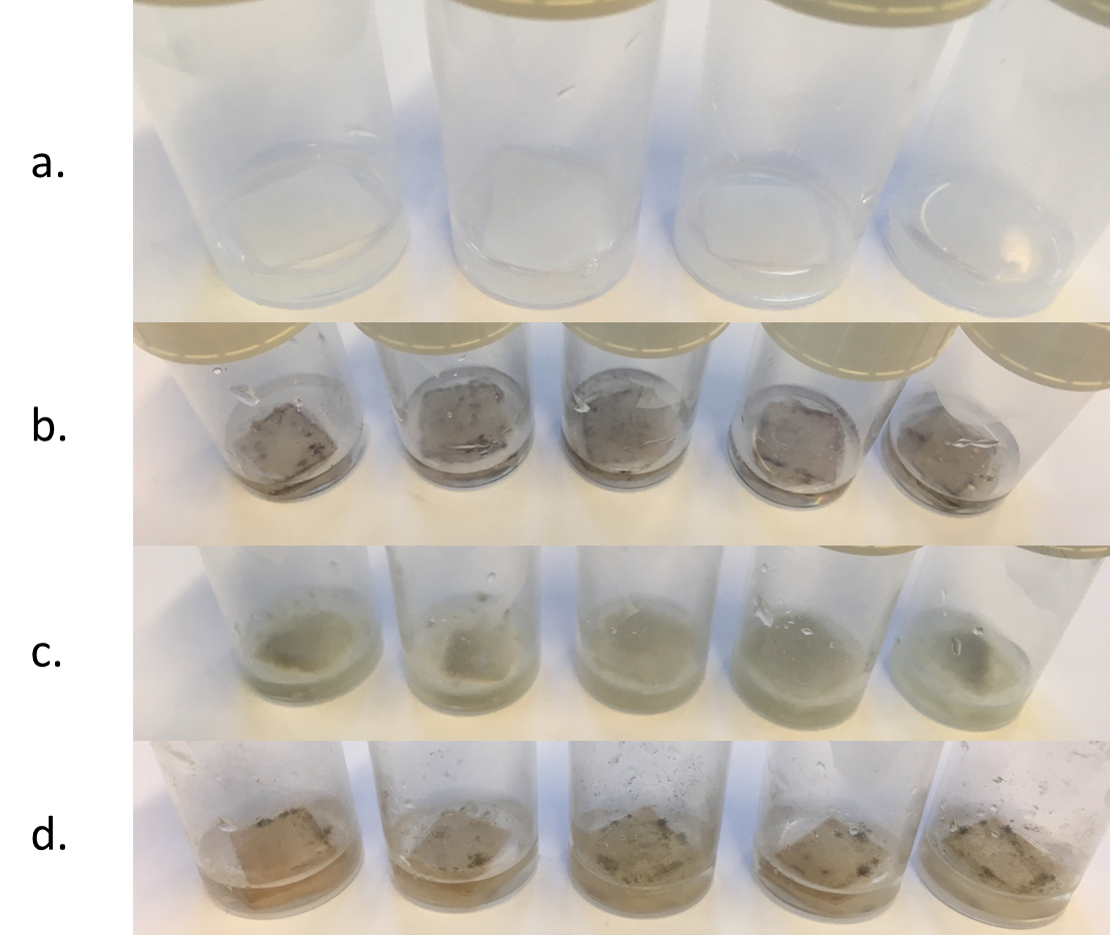

Supplement: Supplementary file 1 — Fig. S1. Evaluation of the fungal growth on the PCL‐based TPU after 2 months of incubation at 30°C. [file MBT2-12-544-s001.png]

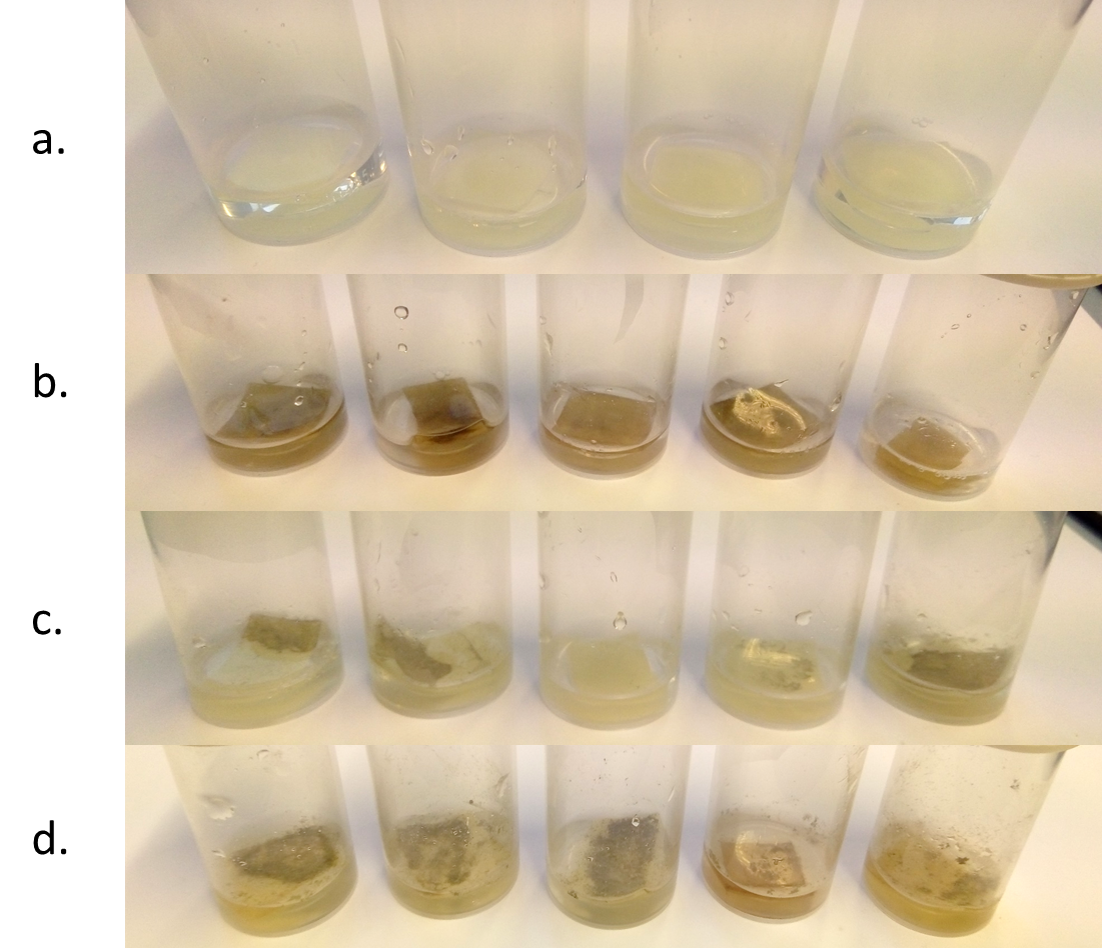

Supplement: Supplementary file 2 — Fig. S2. Evaluation of the fungal growth on the fatty acid dimer‐based TPU after 2 months of incubation at 30°C. [file MBT2-12-544-s002.png]

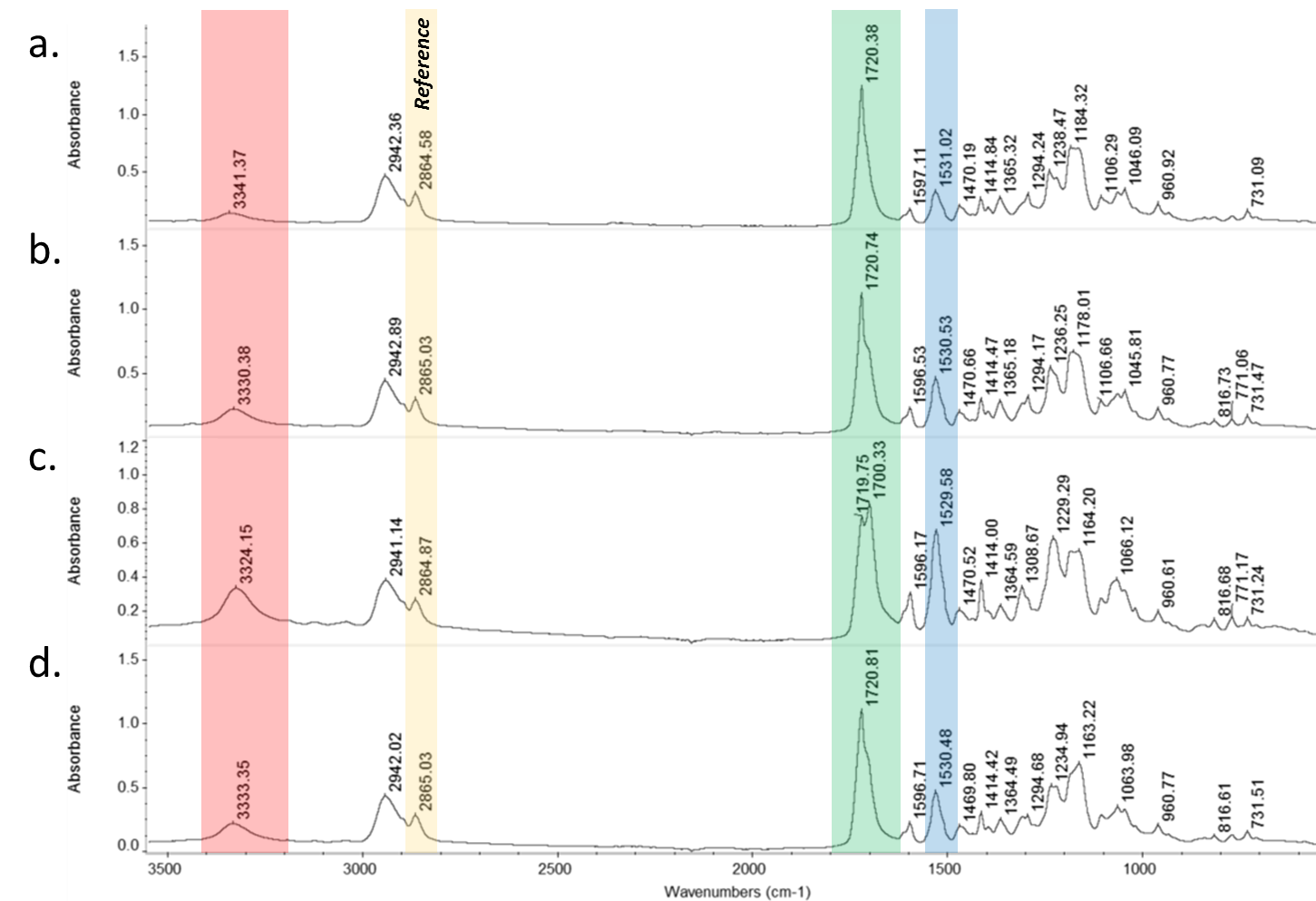

Supplement: Supplementary file 3 — Fig. S3. Absorbance spectra of FTIR analysis for PCL‐based TPU incubated 2 months at 30°C. [file MBT2-12-544-s003.png]

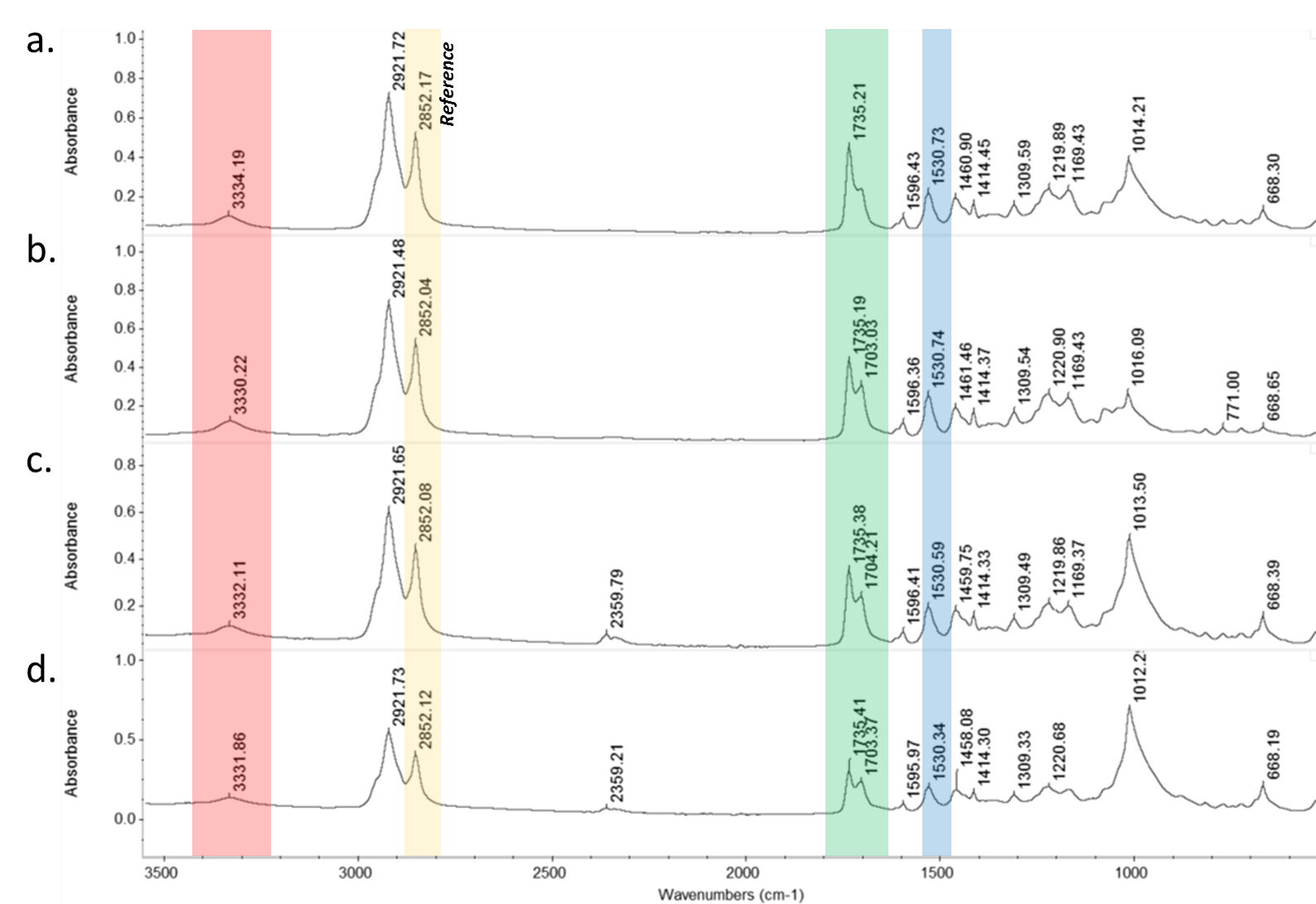

Supplement: Supplementary file 4 — Fig. S4. Absorbance spectra of FTIR analysis for fatty acid dimer‐based TPU incubated 2 months at 30°C. [file MBT2-12-544-s004.png]

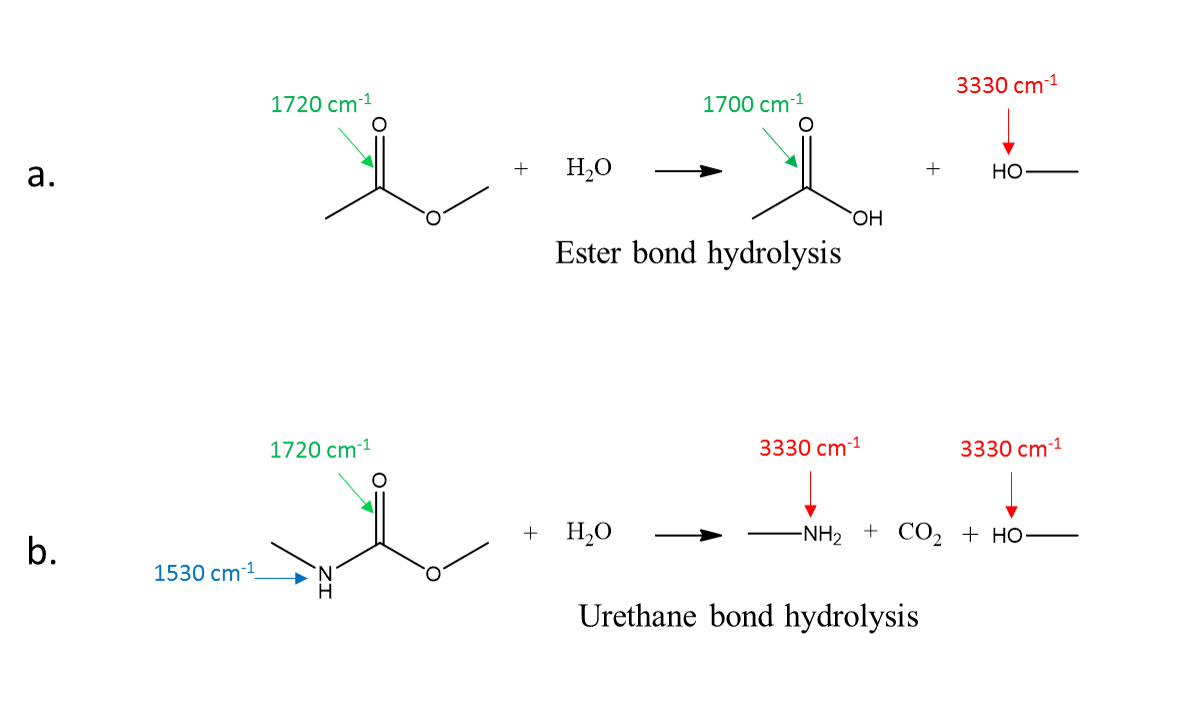

Supplement: Supplementary file 5 — Fig. S5. FTIR signals attribution. [file MBT2-12-544-s005.png]

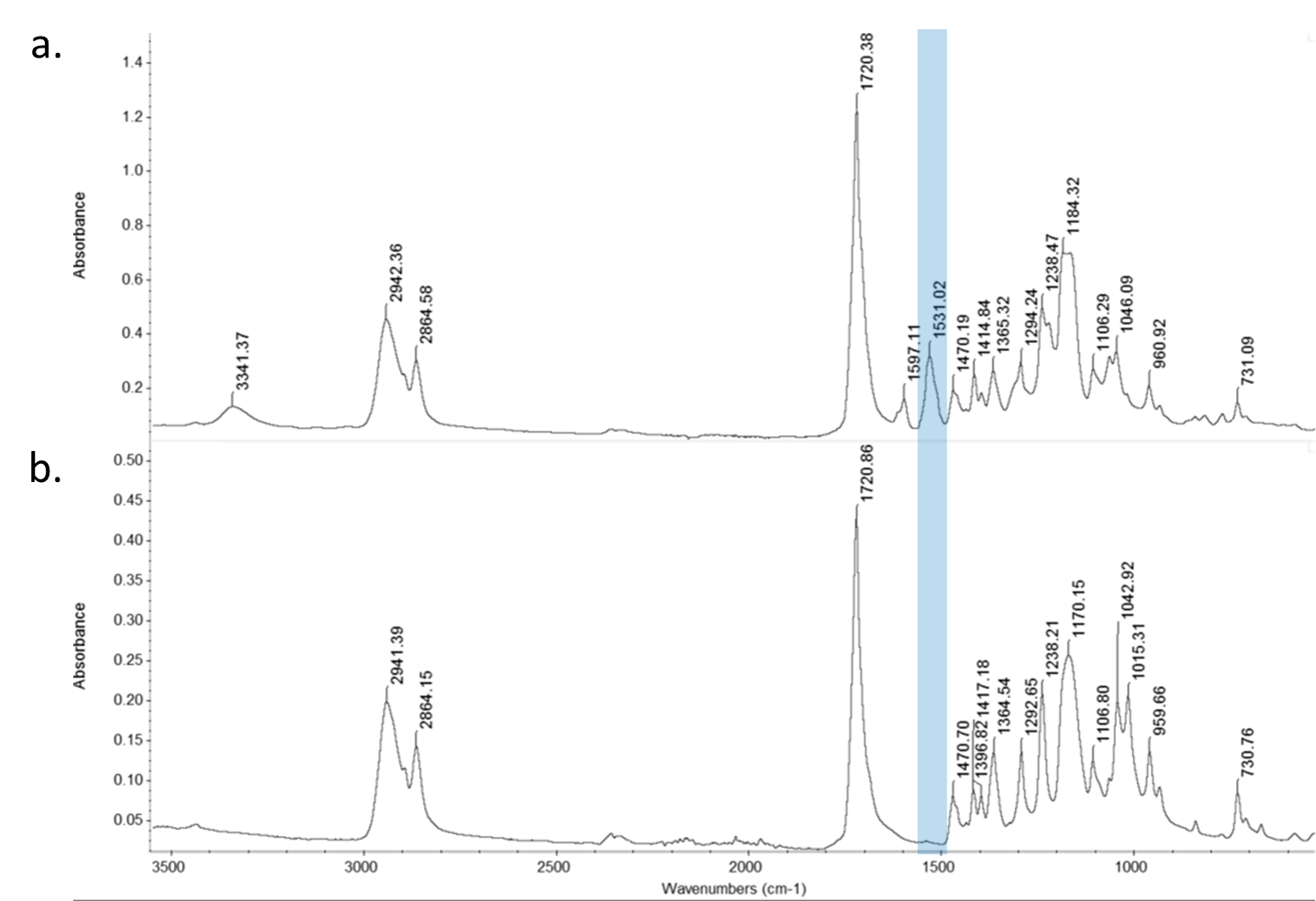

Supplement: Supplementary file 6 — Fig. S6. Comparison of absorbance FTIR spectra of the PCL‐based TPU and the PCL polyester. [file MBT2-12-544-s006.png]

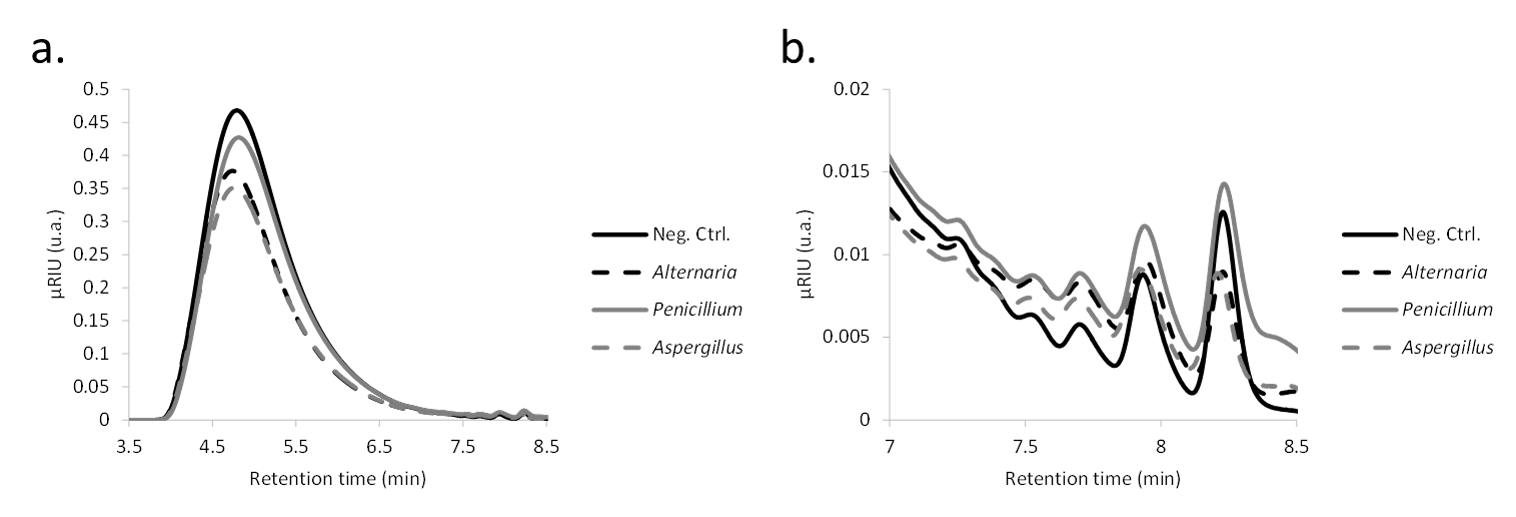

Supplement: Supplementary file 7 — Fig. S7. SEC analysis of the PCL‐based TPU incubated 2 months at 30°C with fungi. [file MBT2-12-544-s007.png]

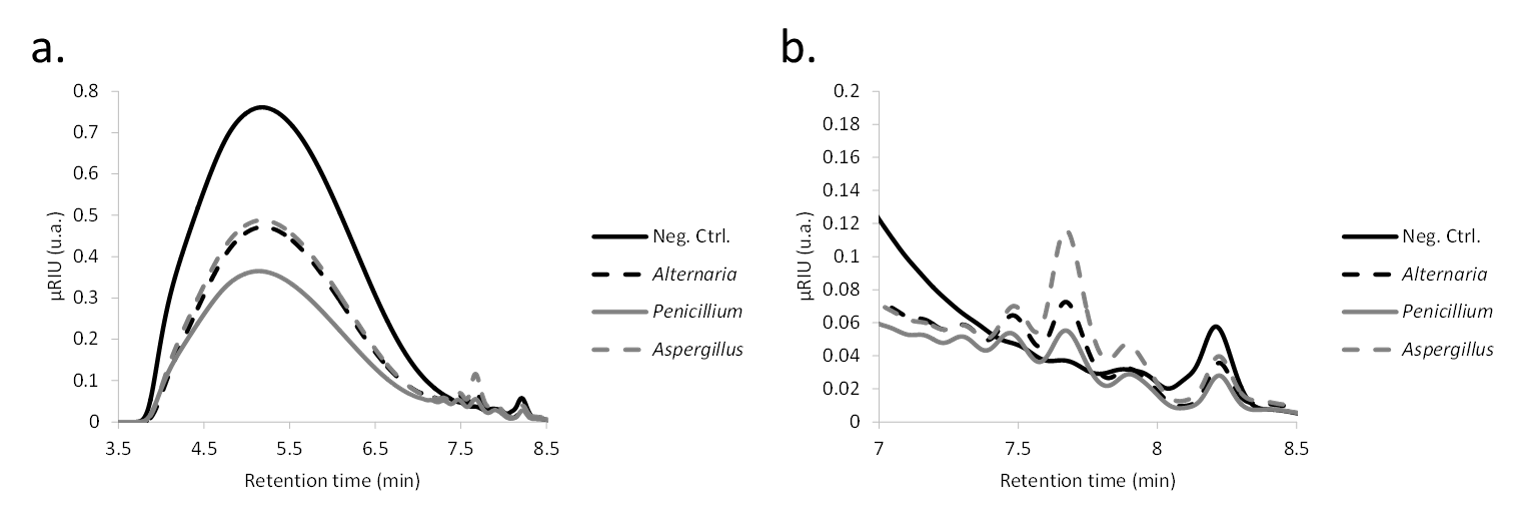

Supplement: Supplementary file 8 — Fig. S8. SEC analysis of the fatty acid dimer‐based TPU incubated 2 months at 30°C with fungi. [file MBT2-12-544-s008.png]
